# Treatment of Wide-Necked Bifurcation Aneurysms: Initial Results with the pCANvas Neck Bridging Device

**DOI:** 10.1007/s00062-018-0680-6

**Published:** 2018-03-19

**Authors:** P. Lylyk, J. Chudyk, C. Bleise, H. Henkes, P. Bhogal

**Affiliations:** 1 Clinica Sagrada Familia, ENERI, Buenos Aires, Argentina; 2 Neuroradiological Clinic, Neurocenter, Klinikum Stuttgart, Kriegsbergstr 60, 70174 Stuttgart, Germany; 3 Medical Faculty, University Duisburg-Essen, Essen, Germany

**Keywords:** Aneurysm, Bifurcation, pCANvas, Wide neck, Stent

## Abstract

**Background:**

Recently, numerous devices dedicated to the treatment of wide-necked aneurysms have become available. We present our initial experience with the pCANvas device and present the technical success rate, clinical outcome and immediate angiographic occlusion rates.

**Objective:**

We sought to determine the efficacy of flow with the pCANvas for the treatment of unruptured intracranial aneurysms.

**Methods:**

We performed a retrospective review of our prospectively collected data to identify patients treated with the pCANvas device between February 2015 and February 2017. The patient demographics, aneurysm characteristics, immediate and delayed clinical and radiographic follow-up data were recorded.

**Results:**

We identified 17 patients (13 female) treated only with the pCANvas device. The average age of the patients was 60.5 ± 13.3 years (range 25–75 years). The average dome width was 7.6 ± 3.2 mm (range 3–15.8 mm), dome height 7.1 ± 3.2 mm (range 3–12.9 mm) and neck width 5.4 ± 3.2 (range 3–16.3 mm). The average aspect ratio was 1.5 ± 0.8 (range 0.6–3.7). At the end of the procedure 15 aneurysms continued complete filling of the aneurysm (Raymond Roy Classification[RRC] 3) with 2 aneurysms showing only filling of the neck of the aneurysm (RRC 2). Early follow-up angiography was available for 16 patients and at this stage 11 aneurysms showed persistent and complete filling of the aneurysm (RRC 3), 5 aneurysms showed complete occlusion of the aneurysm (RRC 1) and 7 aneurysms underwent repeat treatment with coiling.

**Conclusion:**

The early results on the use of the pCANvas are promising; however, longer term follow-up and larger studies are required.

## Introduction

Bifurcation aneurysms are a challenging entity to treat and a wide variety of different coiling methods, including balloon-assisted, stent-assisted and the waffle cone technique have been used to treat these aneurysms [1–7]. Dedicated devices have recently entered the market that have been designed specifically to assist in the treatment of these aneurysms and include the pCONus1 and pCONus2 (phenox, Bochum, Germany), the PulseRider® (Pulsar Vascular, Los Gatos, CA, USA), and the eCLIPs™ device (Evasc Neurovascular Enterprises, Vancouver, BC, Canada). These devices share the common feature of providing extra coverage at the aneurysm neck to prevent coil prolapse into the parent vessel. Intrasaccular devices such as the WEB™ (Sequent Medical, Aliso Viejo, CA, USA) represent an alternative technique [8–10].

**Fig. 1:**
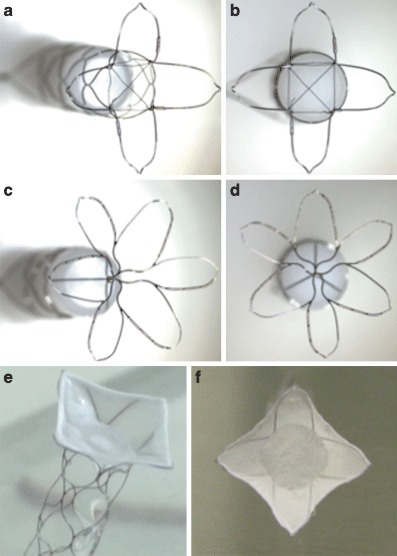
The original pCONus1 (**a** and **b**) device has a distal crown and crossing polyamide fibers that are designed to improve coil retention in the aneurysm. The pCONus2 (**c** and **d**) has six petals with radio-opaque markers on each of the petals to allow accurate positioning within an aneurysm. The design of the pCONus2 allows for the crown to articulate and hence accommodate steep angles between the parent vessel and the aneurysm and the greater metal coverage centrally within the device is designed to assist aneurysm coiling and minimize the chance of coil protrusion into the parent vessel. The pCANvas (**e** and **f**) has a complete impermeable membrane covering the distal end of the stent structure as well as the distal crown. There are four radio-opaque markers on the distal crown to allow accurate placement of the device in the aneurysm neck

The pCANvas is a third-generation device comprised of a laser-cut electrolytically detached stent shaft with a distal crown of petals. Unlike the pCONus1 and pCONus2 devices the distal petals are covered in a biocompatible membrane that acts to inhibit flow into the aneurysm as well as prevent coils being displaced into the parent vessel. There are four radio-opaque markers on the distal crown to allow accurate placement of the membrane-covered crown at the aneurysmal neck. This is the first neck bridging neurovascular device to incorporate this feature. The membrane is impermeable to blood flow; however, it can be easily punctured with standard micro-guidewires and microcatheters to allow additional coiling of aneurysms (Fig. 1).

In this study we present our initial experiences with the pCANvas device and present the technical success rate, complication rate, clinical and angiographic follow-up rate. This is the first publication to present clinical data on the use of the pCANvas device.

## Materials and Methods

We retrospectively reviewed our database of prospectively collected data to identify all patients who were treated with the pCANvas device between February 2015 and February 2017. We excluded patients who were treated with adjunctive coils at the same sitting as well as patients treated for recurrent aneurysms. Patients treated in the acute phase after subarachnoid hemorrhage (SAH) were excluded from the analysis. All patients gave informed consent and the use of the pCANvas device was at the discretion of the operator. All procedures were carried out with the patient under general anesthesia. All patients received dual antiplatelet therapy (aspirin 75 mg daily and clopidogrel 75 mg) started 7 days prior to the planned treatment. The effectiveness of the antiplatelet regimen was tested using the Multiplate® analyzer (Roche, Basel, Switzerland) within 24 h of the planned procedure. In patients who demonstrated resistance to clopidogrel, ticagrelor (180 mg) was used as a substitute. The post-procedural antiplatelet regimen consisted of clopidogrel/ticagrelor continued for 6 months following treatment and aspirin continued for life.

A standard 6 Fr right common femoral approach was used for all cases. Patients were fully heparinized with a 5000 IU bolus dose of heparin and repeated bolus doses of heparin to maintain the activated clotting time (ACT) at 2–2.5 times normal. Rotational angiography was used to accurately ascertain the anatomy of the aneurysm, afferent artery and any vessels derived from the neck of the aneurysm. This was also used to correctly size the pCANvas device to both the afferent artery and the aneurysm neck/base.

### Clinical Events

Any clinical events that occurred in the post-operative period were recorded. A neurological assessment was performed prior to the treatment, after the treatment, at discharge and at follow-up.

### Radiological Assessment

Standard Towne’s and lateral angiographic images were acquired for all patients prior to treatment in addition to rotational angiography in order to determine the most suitable projection to allow visualization of the afferent artery, the aneurysm and any branches that needed preserving. Control angiography was performed at the end of the procedure. Aneurysm measurements were made using standard methods. The maximum fundus width, neck width and height measurements were recorded using standard techniques. Aneurysm occlusion grading was based upon the Raymond-Roy classification (RRC).

## Adherence to Ethical Standards

The retrospective analysis and anonymous publication of the data presented here was approved by the responsible Institutional Review Board.

## Results

We identified 17 patients (13 female) with 17 aneurysms treated with the pCANvas device alone. The average age of the patients was 60.5 ± 13.3 years (range 25–75 years). The aneurysms ranged in size and location with average dome width being 7.6 ± 3.2 mm (range 3–15.8 mm), dome height 7.1 ± 3.2 mm (range 3–12.9 mm) and average neck width 5.4 ± 3.2 (range 3–16.3 mm). The average aspect ratio (dome height to neck width) was 1.5 ± 0.8 (range 0.6–3.7) and the average bottleneck factor (dome width to neck width) was 1.5 ± 0.6 (range 1–3). Of the aneurysms four were midline, five were located on the right and eight were located on the left. Aneurysms were located in both the anterior and posterior circulation with the MCA bifurcation being the most common location (*n* = 10), followed by the AcomA (*n* = 3), ICA bifurcation (*n* = 3), and the basilar tip (*n* = 1). None of the aneurysms were previously treated and all aneurysms were unruptured at the time of treatment. None of the aneurysms were treated with adjunctive coiling at the time of pCANvas implantation. The aneurysm characteristics and demographic data are shown in Table 1.

**Table 1:** Demographic data and baseline characteristics of the aneurysms

Patient number	Gender	Age	Aneurysm Characteristics								pCANvas size (mm)	Adjunctive coiling at time of pCANvas	Intraoperative complications
			Dome height (mm)	Dome width (mm)	Neck width (mm)	Aspect ratio	Bottleneck factor	Laterality	Aneurysm location	Ruptured			
1	F	63	5	8	4	1.3	2.0	L	MCA Bif	*N*	4 × 25 × 6	*N*	*N*
2	F	75	5	10	8	0.6	1.3	Midline	AcomA	*N*	4 × 25 × 8	*N*	*N*
3	F	71	6	5.5	5	1.2	1.1	L	MCA Bif	*N*	4 × 25 × 6	*N*	*N*
4	F	37	7	9	3	2.3	3.0	L	ICA Bif	*N*	4 × 25 × 6	*N*	*N*
5	M	53	10	9	6.5	1.5	1.4	R	MCA Bif	*N*	4 × 25 × 10	*N*	*N*
6	M	68	3	3	3	1.0	1.0	L	MCA Bif	*N*	4 × 25 × 6	*N*	*N*
7	F	55	7	5	3.7	1.9	1.4	L	ICA Bif	*N*	4 × 25 × 6	*N*	*N*
8	F	72	12.9	15.8	16.3	0.8	1.0	R	MCA Bif	*N*	4 × 25 × 12	*N*	*N*
9	F	61	7	9	5	1.4	1.8	L	MCA Bif	*N*	4 × 25 × 8	*N*	*N*
10	F	66	14	11	5	2.8	2.2	Midline	AcomA	*N*	4 × 25 × 6	*N*	*N*
11	M	25	6	8.5	5	1.2	1.7	L	MCA Bif	*N*	4 × 25 × 6	*N*	*N*
12	F	75	11	8	3	3.7	2.7	L	ICA Bif	*N*	4 × 25 × 10	*N*	*N*
13	M	53	7.5	8.5	8.4	0.9	1.0	R	MCA Bif	*N*	4 × 25 × 10	*N*	*N*
14	F	61	4	5	3.5	1.1	1.4	Midline	Basilar Tip	*N*	4 × 20 × 6	*N*	*N*
15	F	59	3	4	4	0.8	1.0	R	MCA Bif	*N*	4 × 20 × 6	*N*	*N*
16	F	70	4.5	3.5	3.5	1.3	1.0	R	MCA Bif	*N*	4 × 20 × 5	*N*	*N*
17	F	65	7	7	6	1.2	1.2	Midline	AcomA	*N*	4 × 20 × 8	*N*	*N*

*MCA* middle carotid artery, *AcomA* anterior communicating artery, *ICA* internal carotid artery, *Bif* bifurcation

## Immediate Angiographic Results

**Fig. 2:**
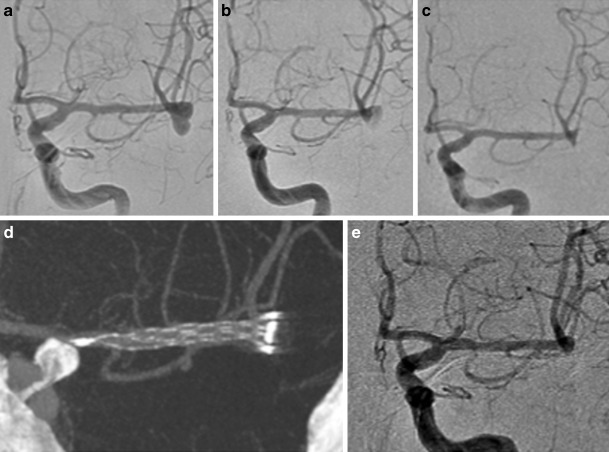
Patient 1 had a lobulated left MCA bifurcation aneurysm (**a**). After implantation of a single pCANvas flow could still be seen within the aneurysm; however, this had decreased (**b**) with no opacification of the aneurysm seen on the 24 h angiography (**c**) or on the intra-arterial (IA) rotational angiography (**d**). The aneurysm remained completely excluded from the circulation on delayed angiography (**e**)

In all cases the pCANvas device was successfully deployed and there were no intra-operative complications. There were no intraoperative aneurysmal ruptures. At the end of the procedure angiography showed that 15 aneurysms had complete filling of the aneurysm (RRC 3) with two aneurysms showing only filling of the neck of the aneurysm (RRC 2). Angiography was repeated in all patients at 24 h and at this stage 13 aneurysms were graded as RRC 3, 2 aneurysms showed no continued filling of the aneurysm (RRC 1; Fig. 2) and 2 aneurysms showed only neck remnants (RRC 2).

## Early Delayed Angiography

**Fig. 3:**
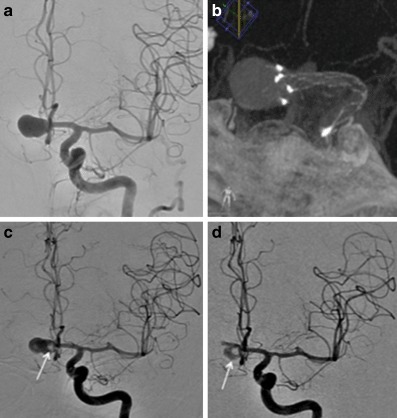
Patient 2 had a large aneurysm originating from the AcomA (**a**) treated with a single pCANvas with the stent shaft tracked into the A1 and terminal ICA (**b**). On follow-up angiography at 4 months, although complete opacification of the aneurysm could be seen there appeared to be thrombus located centrally within the aneurysm and likely adherent to the membrane (**c**, *white arrow*). This central thrombus was more prominent at the 8‑month follow-up angiography alongside some decrease in the overall opacification of the aneurysm (**d**, *white arrow*). We believe that this central thrombus may occur as a consequence to the changes in flow with in the aneurysm caused by the membrane and is covered in more detail in the discussion

**Fig. 4:**
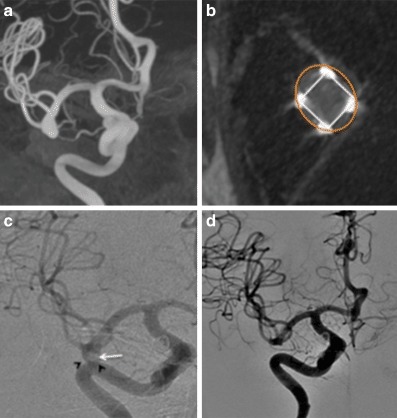
Patient 3 had a right MCA bifurcation aneurysm (**a**). On a “barrel of the gun” view, through the MCA and the aneurysm, the markers of the membrane can be clearly seen and the approximate position of the membrane at the neck of the aneurysm can be estimated by forming a square that connects the four markers (**b**, *white square*). It can also be seen that there is a small area around the membrane that is not covered as the neck and proximal aneurysm form oval shape (**b**, *orange oval*). This may result in flow around the aneurysm with relative stasis of flow centrally behind the membrane that we believe results in the central thrombus. Central thrombus could be seen on the delayed 8‑month angiography (**c**, *white arrow*) with ongoing contrast opacification seen around the central thrombus and adjacent to the wall of the aneurysm (**c**, *black arrow heads*). A small aneurysm neck remnant (RRC 2) was seen at the most recent angiography (**d**)

**Fig. 5:**
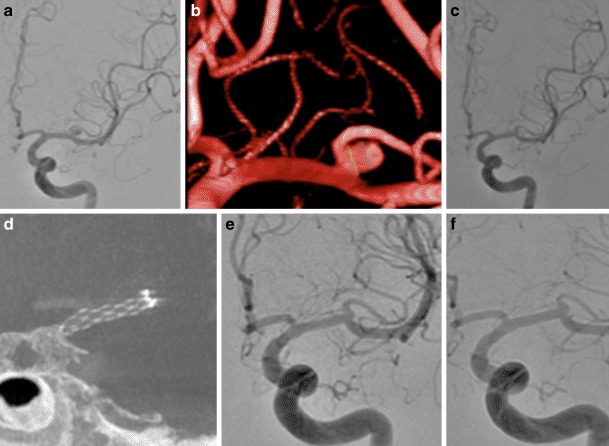
Patient 6 had a small wide-necked aneurysm of the left MCA bifurcation (**a**, **b**) successfully treated with a single pCANvas. A small neck remnant could be seen at the end of the procedure (**c**). Given the small size of the aneurysm the distal crown does not appear to have “flared” open (**d**); however, this relative covering of the membrane at the neck of the aneurysm was sufficient to cause aneurysm thrombosis with complete exclusion of the aneurysm seen on 6‑month (**e**) and 8‑month angiography (**f**)

Early follow-up angiography was available for 16 patients at a median of 6.1 months (range 3–10 months) after the procedure. At this stage 11 aneurysms showed persistent and complete filling of the aneurysm (RRC 3) and 5 aneurysms showed complete occlusion of the aneurysm (RRC 1; Figs. 3, 4 and 5).

## Mid-Term Angiographic Follow-Up

Mid-term angiographic follow-up was available for 14 patients at a median of 18.2 months (range 7–38 months) post-treatment: 3 aneurysms showed complete filling of the aneurysm, 3 aneurysms had neck remnants and the remainder were completely occluded.

## Retreatment

To date, 7 aneurysms have undergone repeat treatment with coiling of the aneurysms after the initial pCANvas procedure and follow-up angiography. In all cases this was because of a failure of the aneurysm to occlude after treatment with the pCANvas alone.

The results are summarized in Table 2.

**Table 2:** Angiographic and clinical follow-up data

Patient number	Immediate post-procedure angiography (RRC)	Initial FU	Outcome of initial FU (RRC)	Initial delayed FU	Outcome of delayed FU (RRC)	Delayed FU	Outcome of delayed FU (RRC)	Intraoperative complications	mRS	Retreatment	Comments
1	3	24 h	1	8 months	1	38 months	1	*N*	0	*N*	–
2	3	24 h	3	8 months	3	17 months	2	*N*	0	*N*	–
3	3	24 h	3	6 months	1	17 months	1	*N*	0	*N*	–
4	3	24 h	3	5 months	3	7 months	3	*N*	0	Y	Coiled after 7 months
5	3	24 h	3	6 months	3	9 months	1	*N*	0	Y	Coiled after 6 months
6	2	24 h	2	6 months	1	8 months	1	*N*	0	*N*	–
7	3	24 h	3	7 months	3	20 months	1	*N*	0	Y	Coiled after 7 months
8	3	24 h	3	4 months	3	28 months	3	*N*	0	Y	Coiled after 4 months FU
9	3	24 h	3	5 months	3	32 months	3	*N*	0	*N*	–
10	3	24 h	3	10 months	3	10 months	2	*N*	0	Y	Device displaced—coiled after 10 months
11	3	24 h	3	6 months	3	22 months	1	*N*	0	Y	Coiled after 6 months
12	3	24 h	1	10 months	1	–	–	*N*	0	*N*	–
13	3	24 h	3	3 months	3	–	–	*N*	0	*N*	–
14	3	24 h	3	3 months	3	20 months	1	*N*	0	Y	Coiled after 10 months FU
15	3	24 h	3	6 months	1	19 months	1	*N*	0	*N*	–
16	2	24 h	2	–	–	–	–	*N*	0	*N*	–
17	3	24 h	3	4 months	3	8 months	2	*N*	0	*N*	–

*FU* follow-up, *RRC* Raymond-Roy Classification, *mRS* modified Rankin Score

## Complications

There were no intraoperative complications and no mortalities. There were no complications in the early postoperative period (<30 days) and all patients were modified Rankin Score (mRS) 0 at the most recent clinic follow-up.

**Fig. 6:**
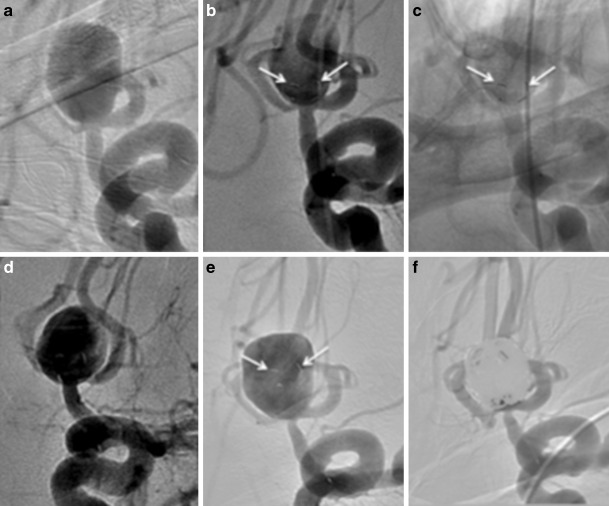
Patient 10 had a large aneurysm of the AcomA (**a**) treated with a single pCANvas device. The markers on the distal crown can be clearly seen at the neck of the aneurysm on the angiography performed at the end of the procedure (**b** and **c**, *white arrows*). On angiography at 24 h the appearance was stable with no significant change in the opacification of the aneurysm (**d**). Angiography performed at 10 months (**e**) showed that the device had migrated into the aneurysmal dome (**e**, *white arrows*). The aneurysm was subsequently coiled with only a small neck remnant at the end of the procedure (**f**)

In a single case (patient 10), at the early follow-up angiography the device had displaced into the aneurysmal sac (Fig. 6). We believe this was due to the water hammer effect of the blood against the membrane as well as the relatively short stent shaft in this case that may not have provided sufficient friction to prevent this distal migration of the device. This did not result in aneurysmal rupture or any other complication. The aneurysm was successfully coiled (RRC 2).

## Discussion

Just under two thirds of all intracranial aneurysms occur at bifurcations [11] and wide necked bifurcation aneurysms pose a particular challenge for treatment via the endovascular approach. In order to deal with this challenging morphology a variety of techniques were developed, including balloon remodeling, waffle-cone stenting, Y‑stenting and T‑stenting. Despite this plethora of different endovascular techniques these aneurysms remain a challenge. Recently several devices aimed specifically at dealing with these aneurysms have been developed. These devices include the PulseRider®, the pCONus1, pCONus2 and the eCLIPs™ device. The pCANvas represents a third-generation device designed to not only prevent coil protrusion into the parent vessel but also to limit blood flow into the aneurysm and hence offer additional aneurysmal protection.

There are several existing neck bridging devices, including the pCONus1, PulseRider®, and the eCLIPs™. Numerous case series have been published detailing the use of the pCONus1 in both ruptured and unruptured aneurysms as well as in conjunction with other devices [12–16]. Gory et al. [17] recently published their 1‑year results on the use of the pCONus1 in wide-necked middle cerebral artery aneurysms. In this series 29 patients had a 12-month follow-up, with an average follow-up of 11.9 months for the whole cohort (range 3–20 months). In total 31 patients did not require retreatment and of the 9 aneurysms that required retreatment, 6 were large (>10 mm) and 7 aneurysms had neck remnants at immediate angiography. There were no new complications seen in the cohort and no complications associated with the retreatment. The authors did not report any cases of in-stent stenosis or covered side branch occlusion. To date there is only a single series published on the pCONus2 device; however, the early results appear promising [18]. The results of the Adjunctive Neurovascular Support of Wide-necked aneurysm embolization and Reconstruction trial (ANSWER), using the PulseRider®device, were also recently published [19]. This was a prospective, non-randomized, single-arm multicenter study conducted at 10 neurovascular centres in the USA. A total of 34 patients were enrolled (29 female) with a mean age of 60.9 years and the majority of the aneurysms were located at the basilar tip (*n* = 27). There were no procedure/device-related deaths and mRS ≤ 2 was achieved in 32 patients (94.1%) at 180-day follow-up. The study reported three intraprocedural complications but none resulted in clinical sequelae. Satisfactory aneurysm occlusion was seen in 79.4% of cases at day 0 and this improved to 87.9% of cases at 180-day follow-up. The eCLIPs™ is an alternative device, which acts as both a flow diverter and neck-bridging device. The recent publication by Chiu et al. [20] documented their experience with this device in 33 patients, 23 of whom have 6‑month follow-up data with 7 patients achieving RRC I occlusion and 10 RRC II occlusion, resulting in 73.9% adequate occlusion. There were 2 acute periprocedural transient ischemic attacks and 2 patients with asymptomatic thrombotic events giving an overall morbidity of 8.7% for those patients with 6‑month follow-up. A further 2 patients had delayed aneurysm-related deaths; however, these were unrelated to the device. The authors reported that in 8 patients the device itself was not deployed and this was for a variety of different reasons including the device not being appropriately oriented , a spasm in the branch vessel, and failure to deploy the device resulting in an failure rate of approximately 24% which may at least be in part be explained by the learning curve.

The pCANvas represents an advancement on the original pCONus1 device and the subsequent second-generation pCONus2 device. The pCANvas device was designed specifically to modify intra-aneurysmal flow with only the eCLIPs™ designed to have similar flow-modifying properties. Given that hemodynamics play an important role in the recanalization of aneurysms [21] and that high shear stress at the aneurysm neck is a risk factor for aneurysm recurrence [22], the natural evolution of neck bridging devices should prevent intra-aneurysmal blood flow. The flow-modifying effects were recently tested in an in vitro silicone model of a basilar tip aneurysm with a blood-mimicking fluid under pulsatile conditions [23]. Intra-aneurysmal flow was compared between the pCONus1, the Solitaire AB (Medtronic, Irvine, CA, USA) deployed in a manner that replicates the waffle cone technique, and the pCANvas. The results of the study demonstrated that none of the devices implanted in the standard technique caused an increase in intra-aneurysmal flow. Both the Solitaire and the pCONus1 caused a similar small decrease in intra-aneurysmal flow, with a decreased intra-aneurysmal flow ratio of 0.96 for the pCONus (*p* = 0.17) and 0.91 for the Solitaire AB (*p* = 0.01). Most interesting and consistent with the design of the pCANvas, there was a large decrease in the intra-aneurysmal flow following deployment of the pCANvas with the preimplantation and postimplantation flow ratio being 0.4 (*p* =5 × 10^−12^).

**Fig. 7:**
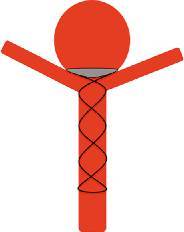
Schematic representation of the pCANvas accurately placed in the base of the aneurysm above the efferent branches. The purpose of the device is to restrict intra-aneurysmal blood flow and hence promote thrombosis with or without adjunctive coiling

Despite the promising bench-side studies our clinical experience suggests that the device cannot be used alone to achieve aneurysm occlusion and must be used as an adjunctive device with standard coils or another form of intrasaccular filling. The device needs to be accurately sized to the diameter of the parent vessel as well as the aneurysm neck and to that end the device is available in a variety of different sizes. Once the device is in situ, standard frontal and lateral projections can be used to accurately position the device in the aneurysm neck away from any daughter branches (Fig. 7).

**Fig. 8:**
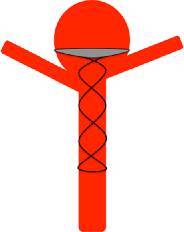
The origin of the efferent branches from the aneurysm play an important role in the accurate placement of the device. The membrane-covered distal crown must be placed just distal to the efferent branch in order to prevent inadvertent occlusion; however, in doing so a small aneurysm remnant may remain. Similar issues may also be encountered with other neck bridging devices

In certain instances, the efferent branches may not be derived from the base of the aneurysm neck and this would then necessitate placing the device deeper into the aneurysm to sit distal to the origin of these vessels (Fig. 8). This positioning of the device would inevitably leave an aneurysm remnant; however, this would also occur with the existing neck bridging devices and could likely only be prevented by Y‑stent or T‑stent assisted coiling of the aneurysm.

**Fig. 9:**
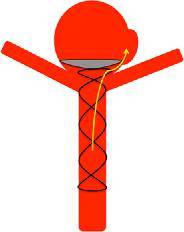
Lobulated aneurysms can result in difficulty sizing the device and lead to poor apposition of the membrane covered crown to the aneurysm wall. This may lead to continued flow around the membrane and into the aneurysm, which could prevent aneurysm occlusion

Similarly, in certain instances the aneurysms may be lobulated and this could result in difficulty in accurately sizing the crown as well as poor opposition of the membrane to the aneurysm wall with resultant flow around the membrane into the aneurysm (Fig. 9).

In a single case the device became displaced into the aneurysm. We believe that this was due to the water hammer effect of the blood against the membrane but principally due to the relatively short stent shaft in the A1 segment. The stent shaft is designed to prevent this from occurring but in certain circumstances, particularly with shorter lengths, the overall friction provided by the stent shaft may not be sufficient to prevent this intra-aneurysmal displacement from occurring; however, we believe that this is unlikely to occur if intrasaccular coiling is concomitantly performed and additionally we would recommend that a pCANvas with as long a stent shaft as possible be chosen to prevent this potential delayed complication.

**Fig. 10:**
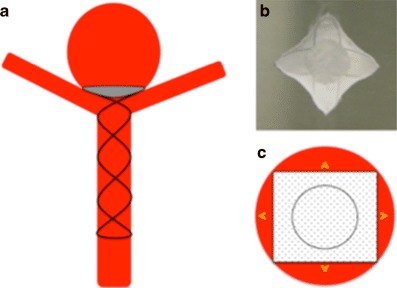
On standard projections the positioning of the pCANvas device may be entirely satisfactory (**a**); however, as the membrane covering is square (**b**) and the aneurysm neck is likely to be circular or at the very least rounded, the membrane is unlikely to completely cover the aneurysm neck and blood flow may well continue entering the aneurysm around the edges of the membrane covering (**c**, *yellow arrows*). This phenomenon can also be seen in Fig. 4b

**Fig. 11:**
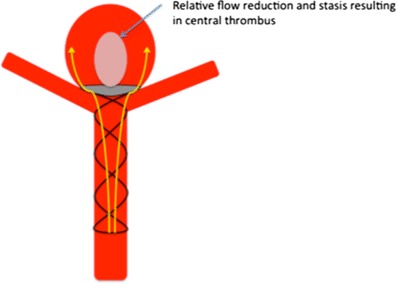
The continued aneurysm flow around the edges of the membrane may result in a reduced zone of blood flow centrally within the aneurysm behind the membrane with continued flow around the wall of the aneurysm. This may result in thrombosis occurring centrally in the aneurysm as was seen in one of our cases. This was seen in several of our cases (Figs. 3 and 4)

Our most important insight with this initial iteration of the device is the shape of the membrane covering the distal crown. On standard projections it may appear that the membrane completely covers the neck of the aneurysm; however, the membrane, which is square will not completely enclose the essentially circular aneurysmal neck (Fig. 4b and 10). This will inevitably result in continued flow into the aneurysm around the edges of the membrane. Although this flow may be reduced it is unlikely to be completely stopped as was suggested by the bench side studies that showed a 60% reduction in intra-aneurysmal flow [23]. This flow may also be predominantly directed around the edges of the aneurysm with relatively decreased flow centrally (Fig. 3c, 3d, 4c and 11) and this may cause thrombosis of the aneurysm to occur centrally, just behind the central portion of the membrane, as was seen in one of our cases.

**Fig. 12:**
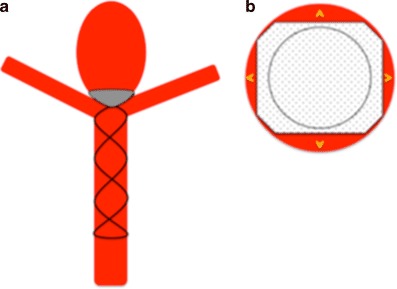
The central portion of the covered membrane, which is circular and conforms to the diameter of the stent shaft, occludes a greater proportion of the aneurysm neck (**a**). This will then result in a smaller area for membrane endoleak around the edges of the square membrane and likely a higher chance of aneurysm occlusion (**b**). This is similar to the situation seen in Fig. 5 where the membrane was kept relatively constrained; however, there was likely sufficient coverage at the neck to occlude the aneurysm

We believe that this “perimembrane endoleak” is principally related to the difference in the size of the parent vessel and the aneurysm neck and that if the neck of the aneurysm is close to the size of the parent artery there will be significant impedance to flow into the aneurysm as well as better conformation of the distal membrane to the shape of the aneurysm neck. Furthermore, as the distal end of the stent shaft is also covered by the membrane and is circular, if the neck of the aneurysm is close to the size of the parent vessel and hence the stent shaft, the coverage will be significantly greater and there will likely be a reduced risk of the aforementioned membrane endoleak (Fig. 12).

Overall the effects of these changes on intra-aneurysmal flow are difficult to predict without further studies. Based on our initial experience we feel that the device should not be used alone and concomitant intrasaccular coiling should be performed to ensure adequate occlusion of the aneurysm and prevention of complications such as distal migration of the device.

Our study has several limitations inherent to a retrospective study as well as the fact that the sample size is relatively small. Long-term results on aneurysm occlusion will require further study and larger prospective studies are required and further studies detailing the use of the device as an adjunct to coiling and in the acute phase are also warranted.

## Conclusion

The pCANvas device is the first neck-bridging device to incorporate a membrane designed to alter intra-aneurysmal flow dynamics. Based on our initial experience the device appears safe; however, we recommend the device is used with adjunctive coiling of the aneurysm. Larger studies and long-term follow-up are required to assess the stability of aneurysm occlusion and long-term safety profile of the device.
